# Living with what is lost: reframing emotional life with diabetes through an integrative perspective on loss and grief

**DOI:** 10.3389/fcdhc.2026.1771568

**Published:** 2026-05-08

**Authors:** Bryan Cleal, Linxi Mytkolli, Kristoffer Bastrup-Madsen Marså, Mette Due-Christensen, Dan Grabowski, Jonathan Garfinkel, Carlo Leget, Mai-Britt Guldin

**Affiliations:** 1 Copenhagen University Hospital, Steno Diabetes Center Copenhagen, Herlev, Denmark; 2 Diabetes Action Canada, Toronto, ON, Canada; 3 Department of Public Health, University of Southern Denmark, Odense, Denmark; 4 Department of Modern Languages and Cultural Studies, University of Alberta, Edmonton, AB, Canada; 5 University of Humanistic Studies, Utrecht, Netherlands; 6 Center for Grief and Existential Values, Aarhus, Denmark; 7 Research Unit for General Practice, Institute for Public Health, Aarhus University, Aarhus, Denmark

**Keywords:** diabetes distress, diabetes self-management, grief/loss, lived experience (of the illness), person-centered, psychosocial adaptation

## Abstract

Over the past three decades, the concept of diabetes distress has played a central role in legitimising the emotional burdens of living with diabetes without pathologising them. Diabetes distress has helped foreground the frustrations, worries, and exhaustion associated with the ongoing demands of self-management and interactions with healthcare systems, and it has provided an important counterweight to purely biomedical models of care. However, while clinically useful, diabetes distress primarily captures emotional responses linked to burden, effort, and perceived threat. It may not fully encompass the quieter, cumulative, and more existential dimensions of emotional life that unfold across the long course of living with diabetes. In this conceptual article, we introduce loss and grief as a complementary lens for understanding these aspects of experience. Drawing on the Integrative Process Model of Loss and Grief (IPM), originally developed within bereavement research, we explore how living with diabetes involves ongoing and often ambiguous losses that affect bodily trust, identity, social participation, imagined futures, and meaning. The IPM conceptualises grief as a dynamic, integrative process unfolding across five interrelated dimensions: physical, emotional, cognitive, social, and spiritual. Rather than treating grief as a time-limited response to a discrete event, the model emphasises adaptation to cumulative and enduring forms of loss, making it particularly relevant to chronic illness. We do not propose grief as an alternative to diabetes distress. Instead, we argue that distress and grief represent overlapping but distinct perspectives on the same lived reality. Diabetes distress foregrounds the pressures and emotional load of self-management, while a grief-informed perspective highlights processes of adaptation, meaning-making, and identity renegotiation over time. Placing these perspectives together allows for a more textured understanding of emotional life with diabetes, including experiences that may not register in screening tools or routine clinical encounters.

## 1 Introduction

Diabetes, whether type 1 or type 2, remains an incurable and potentially progressive condition with serious complications. This inevitably affects not just the biomedical profile of the person diagnosed, but their broader emotional, social, and existential life. Over the past three decades, there has been a growing awareness that a biomedical approach to the treatment of diabetes and its sequelae can only take us so far and that the psychological and social consequences of diabetes are ineffably entwined with the biomedical (1, 2).

One important development in this process has been the emergence of the concept of diabetes distress. Diabetes distress refers to the feelings that arise from the relentless demands of self-care, fears of complications, and interactions with healthcare systems (3–5). It is common and expected, and its emergence has helped position emotional suffering within diabetes care in a way that does not focus upon psychiatric labels (6–9). Its emergence and ever-growing legitimacy (10, 11) have also helped challenge overly biomedical or pathologising accounts of emotional life with diabetes and provided a valuable language for acknowledging patients’ experiences.

Yet despite its clinical utility, the concept of diabetes distress may not fully capture the quieter, more diffuse, or existential forms of emotional difficulty that can play out over the long course of life with diabetes. People with diabetes experience not only distress tied to specific burdens, but also subtler and often unspoken feelings; the erosion of spontaneity, changes in identity, a shrinking of imagined futures, or an intangible sense of unease (12, 13). These are not necessarily dramatic or pathological states, but forms of loss, with accompanying grief, that unfold across the everyday, reshaping how people inhabit their lives.

In this article, we seek to build on the foundation provided by current diabetes distress research by introducing a broader conceptual perspective, drawing on the *Integrative Process Model of Loss and Grief* (IPM) (14). The IPM, developed in the context of bereavement research, offers a framework for understanding how people experience and integrate loss and grief over time, not only in response to death, but also in relation to ambiguous, cumulative, and ongoing forms of loss. The model emphasises that grief is not a time-limited or linear process, but a dynamic and integrative one, unfolding across multiple domains of life. It also recognises that some losses are difficult to articulate or fully acknowledge, particularly when they are chronic, incremental, or socially unrecognised. Although the IPM is used in areas such as dementia, caregiving, and cancer research, it has yet to be meaningfully applied within diabetes research. We explore how this model may help us better attend to the subtle, complex emotional terrain of living with diabetes.

The aim of this paper is to develop a dimensional framework for understanding experiences of grief and loss in adults living with diabetes, foregrounding relational, embodied, and identity-related aspects that are often under-theorised in psychosocial diabetes research. Our aim is not to propose grief as an alternative to diabetes distress or to recast familiar emotional burdens in a new language. Rather, we view distress and grief as overlapping but distinct perspectives that draw attention to different aspects of the same lived experience. Diabetes distress describes the pressures, frustrations, and emotional burdens tied to the daily work of managing diabetes, while a grief-informed perspective draws attention to what diabetes alters, diminishes, or makes uncertain across the longer arc of a person’s life. Distress foregrounds struggle, effort, and the sense of being overwhelmed. Grief foregrounds the work of adapting to change, of integrating what has been lost, and of renegotiating identity and meaning. Placing these perspectives together allows us to see more of the terrain and to recognise forms of emotional experience that do not always register in screening tools or clinical encounters (See **Table 1**).

**Table 1:** Differentiating diabetes distress and integrative process model perspectives.

Feature	Diabetes distress (Fisher & Hessler)	IPM (Guldin & Leget)
Nature of Response	Emotional burden linked to diabetes tasks and threats	A dynamic response to multiple, cumulative, material and symbolic losses
Clinical Framing	Measured, stratified, and addressed via structured tools	Viewed as personal, meaning-laden, and inherently existential
Duration	Episodic or chronic, influenced by events and management	Fluctuating, lifelong potential for reactivation and transformation
Intervention Focus	Reduce distress to improve self-management and QoL	Support adaptation, meaning-making, and relational integration, finding balance
Role of Clinician	Address distress directly, often through structured dialogue	Bear witness to loss, enable narrative exploration and new meaning, support to regain balance

As one of the authors (LM) reflected in a presentation at the Advanced Technologies & Treatments for Diabetes (ATTD) conference in 2025: “I grieve my body, I grieve a system that was not built for me. I grieve the future I have to reimagine constantly. Patient engagement is grief work”. This statement reflects a lived recognition that patient engagement itself is a process of loss and recognition. In diabetes, grief is not confined to the moment of diagnosis, nor complication, but extends to the slow and persistent work of rebuilding trust in one’s body, in health systems, and in the communities that surround care. Engaging with patients, clinicians, and researchers through this lens invites all to see participation not simply as contribution, but as an ongoing act of meaning-making and repair.

### 1.1 Diabetes distress: clarifying burden, obscuring loss?

Diabetes distress has been validated in numerous studies and is not a comorbid disorder or a pathopsychological condition, but simply a consequence of having diabetes (9, 15–17). There is, moreover, a well-documented association between diabetes distress and an increased risk of dysglycemia, though distress may also be present among individuals with diabetes who appear to have well-managed biomedical parameters (18). As this indicates, there is a clear and compelling case that measuring levels of distress and incorporating a focus on it in diabetes care is important.

Fisher et al. (16) offer a practical guide for addressing diabetes distress in clinical settings. They outline five core steps: 1) assess distress systematically, 2) focus on feelings, beliefs, and expectations, 3) help patients gain perspective, 4) develop a concrete plan, and 5) follow up. These steps anchor psychosocial care in diabetes within a structured, actionable framework and reflect a shift toward more person-centered approaches that encourage emotional dialogue, normalize emotional responses, and create space for reflection.

The concept of diabetes distress has been central to articulating the emotional dimension of life with diabetes and has advanced understanding of the worries, frustrations, and burdens that accompany its management and medical demands. Its clinical uptake has brought much needed attention to the everyday challenges that shape psychological wellbeing in diabetes. Leading researchers in this field have also emphasized that addressing distress requires more than identification. Fisher et al. also note that while screening and scoring are important, meaningful care depends on clinicians’ ability to engage in person centered dialogue, attend to emotional nuance, and support processes of meaning making (16). Their model includes actions such as “acknowledging and labeling feelings” and “normalizing” reactions, reflecting an awareness that emotional responses to diabetes are deeply contextual and not easily captured by scales alone.

Yet as the focus on identifying and addressing distress has become more formalized in clinical contexts, the emphasis has tended to fall on measurable indicators of burden or impairment. This clinical drift can make it harder to recognize other facets of the emotional experience of diabetes, such as loss, absence, or longing, that are less amenable to screening but no less significant. Attending to grief and loss offers a way to extend the distress perspective toward these dimensions, highlighting how people navigate what diabetes alters, not only what it demands. Since living with diabetes, like all chronic conditions, can be characterized as a form of “living loss”, (19) the framework of loss and grief may provide a conceptually coherent lens through which to interpret its emotional and existential dimensions.

## 2 The integrative process model of loss and grief

Research on grief has traditionally focused on bereavement, with much of it conducted within the field of palliative care. In 2023, the Integrative Process Model of Loss and Grief was published (20), followed in 2024 by the book Loss, Grief, and Existential Awareness- An Integrative Approach (14). Both these publications present a novel framework for understanding loss and grief: The integrative process model. What characterizes the IPM is that its development draws upon a broad number of scientific fields, such as philosophy, psychology, and biomedicine, to apprehend the physiological, psychological, and social processes occurring in a grieving person (14, 20). In the IPM, grief is understood as a fundamental human experience. Everyone experiences multiple losses and grief throughout life. Grief is a personal experience tied to loss, which may include the loss of employment, physical abilities, health, or spontaneity. Grief is deeply personal and difficult to compare; what one person perceives as a profound loss, another may experience as a minor inconvenience. The intensity and experiences of grief are shaped by the depth of love and attachment to what has been lost and to the weight and significance of anticipated futures. Understanding grief is thereby simultaneously an attempt to understand love or meaning (14, 20).

Another central idea in the IPM is that grief is not simply an emotional response to loss; it is a transformative force. When we lose something or someone dear to us, it changes us. This transformation is not necessarily welcome or redemptive; the IPM makes no claim that grief is a positive experience or that it inevitably leads to personal growth. Grief can be overwhelming, chaotic, or even destructive. Yet even destruction is a form of change. In this view, grief is part of how we are reshaped by what we lose.

Importantly, the IPM does not treat grief as a problem to be solved. Rather, it sees grief as calling for a new kind of balance, for a way of living that integrates the loss into a changed, ongoing life. This emphasis on ongoing adaptation, as opposed to resolution, makes the model especially relevant to experiences of chronic illness, where loss may not be tied to a single event, but unfold gradually, recur unpredictably, and require continual reorientation.

**Figure 1:**
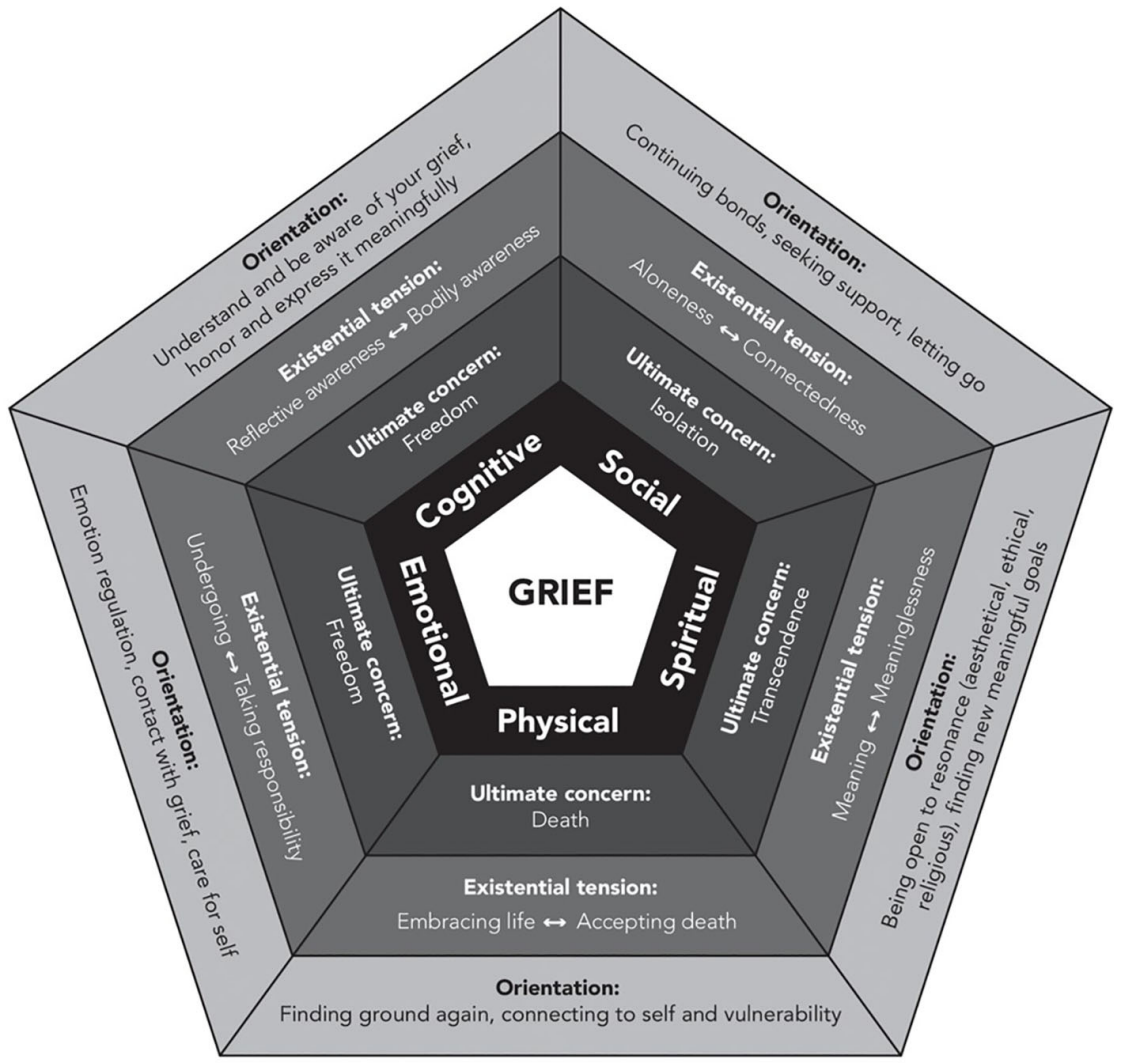
The Integrative Process Model of Loss and Grief (IPM), depicting grief as a dynamic, multidimensional process unfolding across five interrelated domains: physical, emotional, cognitive, social, and spiritual. Each dimension is characterised by an ultimate concern, an existential tension, and a corresponding orientation for adaptation. Together, the model provides a conceptual framework for understanding how individuals engage with and integrate experiences of loss over time.

At the core of the IPM is the idea that grief unfolds across five interrelated dimensions: physical, emotional, cognitive, social, and spiritual (see **Figure 1**). These dimensions are not arbitrary, they reflect core aspects of human experience that are typically affected by significant loss. Each dimension is linked to an “ultimate concern” (a fundamental human need or fear), an “existential tension” (the ambivalence or inner struggle stirred by the loss), and an “orientation” (a potential way of responding or adapting). Together, these dimensions offer a holistic map of the terrain in which grief is lived and navigated. While analytically distinct, they are experienced as deeply intertwined. The terms ‘ultimate concern’ and ‘existential tension’ draw on the work of existential therapist Irvin Yalom (21).

A defining feature of the IPM is its understanding of grief as an ongoing process rather than a time-limited phase to be endured in the service of closure. Grief is not something that can be resolved once and for all, especially when the losses in question are cumulative, ambiguous, or ongoing, as is often the case in chronic illness. In the context of diabetes, this perspective helps illuminate how people live with continuing forms of loss, such as changes in bodily certainty, autonomy, or social identity, that accompany the condition over time. The model does not depict reactions or symptoms of grief in a clinical sense, but instead highlights the challenges and tensions that emerge in each domain of life, inviting reflection, adaptation, and meaning making across the course of living with diabetes.

While the framework presented here is not diagnosis-specific, we attend explicitly to how dimensions of loss, vulnerability, and bodily trust are lived differently across diabetes types, shaped by distinct illness trajectories, temporalities, and social meanings. Likewise, the framework is applied here exclusively with adults living with diabetes in mind; applying it to children or adolescents would require a different, explicitly developmental lens, given differences in cognitive, emotional, and narrative capacities across the life course.

### 2.1 IPM & diabetes: the physical dimension

In the IPM, the physical dimension refers to the bodily consequences of grief and the stress responses triggered by loss. Grief activates physiological stress systems, including the hypothalamic-pituitary-adrenal (HPA) axis, leading to elevated cortisol levels and other neuroendocrine and immune changes (20). These responses may manifest as sleep disturbances, fatigue, muscle tension, loss of appetite, somatic complaints, or heightened inflammatory markers (14, 22, 23). Although often transient, such physical reactions reflect the embodied nature of grief and the ways in which loss can disrupt physical equilibrium.

In diabetes, physical responses to stress and emotional burden are particularly salient, not least because stress is closely tied to blood glucose fluctuations (24, 25). Many people with diabetes report challenges related to eating, whether through disrupted appetite, emotional eating, or complex feelings about food and loss of control (26, 27). Among individuals with type 1 diabetes and parents of children with type 1 diabetes, mealtimes can become a source of intense stress, guilt, or vigilance, especially when framed in relation to long-term complications or immediate risks with diabetes (28–30). Likewise, disturbed eating patterns, including binge eating, are significantly higher among people with type 2 diabetes than in the general population (31, 32) In both instances, eating behaviours can be both a direct source of grief and, in some cases, a physical manifestation of a grief response (33).

Although the IPM tends to frame the physical dimension as a transient response to a discrete loss, life with diabetes challenges this framing. People with diabetes often live with ongoing, anticipatory, or cumulative physical losses. We return to this point in the discussion, where we reflect on how the physical dimension of grief might be reimagined in the context of chronic, progressive illness.

Finally, while the IPM focuses on the individual, it is worth noting that physical manifestations of grief, such as disturbed sleep or heightened anxiety, can also appear among caregivers. In diabetes, this is particularly evident in parents who experience chronic worry about nocturnal hypoglycemia in their children and partners to people living with type 1 diabetes (34–36).

#### 2.1.1 Ultimate concern – death (survival)

For many individuals with diabetes, the fear of physical complications and, ultimately, death constitutes a significant aspect of life (37). The fear of hypoglycemia is well-known amongst persons with type 1, their partners, and parents of children with diabetes (38, 39) and fear of death is reported for both adolescents, parents of children with diabetes, and adults with type 1 and 2 diabetes (40–42). The ultimate concern for both people with diabetes and the healthcare professional is disease progression, blood glucose fluctuations, complications, and death (9, 37, 43).

#### 2.1.2 Existential tension – embracing life – accepting death

In the IPM, the physical dimension is linked to an existential tension between embracing life and accepting death. After a significant loss, people often struggle to reconnect with their body and sense of self (37, 44). The body may feel alien, broken, or out of balance. It is no longer the familiar ground of being, but a site of disruption and vulnerability. In this dimension, grief is not only about what has been lost, but about learning to live in a body that is changed, is continually changing, and marked by the awareness of mortality.

In diabetes, this existential tension is felt acutely. The need for constant self-monitoring (45), the material imposition of medical devices (46), and the ever-present possibility of complications (5) can unsettle the sense of bodily trust and evoke a deep disappointment in a body perceived to have failed. Here, the existential tension between embracing life and accepting death plays out not only in dramatic moments but in the quotidian rhythms of self-care. Like grief, therefore, diabetes calls for a process of reconnection, not to regain full control, but to find a sense of balance.

The IPM names a virtue that can help foster this reconnection. Humilitas is a humility rooted not in resignation, but in the recognition of one’s limitations, vulnerability, and need for others. In the context of diabetes, this humility can be understood as the capacity to live with being situated and limited and, to let go of the ideal of complete mastery, and to find meaning in a body that is both fragile and capable. For health care professionals, humilitas supports the recognition that a focus on glycemic outcomes, for example, can sometimes overshadow the realities of a person’s life. This is not a form of giving up. Rather, it is a way of moving forward with care and accord for the rhythm of daily life.

#### 2.1.3 Orientation in the physical dimension with diabetes

In the IPM, the orientation connected to the physical dimension is about regaining a foothold after the shock of loss (14). This involves regulating the nervous system and re-establishing routines of sleeping, eating, and daily life. It is through these physical patterns that a sense of safety and continuity begins to return.

In the context of diabetes, the existential tension may be experienced less as embracing life versus accepting death, and more as embracing life while accepting illness and its limitations. Physical routines, especially around meals, exercise, rest, and bodily awareness, are essential not only for blood glucose management but also for restoring a sense of groundedness and agency.

This daily attunement to the body’s signals, needs, and limits reflects what the IPM describes as a striving to reconnect with the body in a changed reality. It is here that the model begins to suggest clinical relevance, understanding routines not just as self-management strategies, but as embodied practices of adaptation and emotional integration, which are fundamental to living well with chronic illness.

### 2.2 IPM & diabetes: the emotional dimension

In the IPM, the emotional dimension of grief encompasses a wide range of feelings, including despair, distress, guilt, anger, hostility, longing, yearning, and more (14). These emotions may emerge in waves or lie just beneath the surface, shaping how people engage with themselves and the world after loss. Central to this dimension is the recognition that emotional life is not fully within our control. We cannot choose our feelings at will, but we are not helpless in the face of them either.

The IPM accentuates the importance of emotional flexibility, that is, the ability to stay present with painful emotions when needed, and to set them aside when they threaten to overwhelm. This is not about suppressing or denying feelings, but about cultivating the capacity to shift between engagement and containment. Regaining a sense of daily life without emotional flooding is a key part of adapting to loss.

This balance is particularly relevant in the context of diabetes. Emotional responses to the relentlessness of self-care, fears about the future, or perceived failure can lead to episodes of burnout, where emotional distress becomes so overwhelming that individuals may disengage from diabetes management altogether (47). While diabetes distress is often chronic and backgrounded, burnout is more acute and episodic, yet the two are intimately linked. Emotional overload, especially when paired with a sense of shame or moral failure, can erode well-being and self-efficacy (48).

Research suggests that emotion regulation difficulties contribute both to diabetes distress and to poorer clinical outcomes (18, 49). But the emotional challenges of diabetes are often framed in behavioural or cognitive terms rather than as manifestations of grief. By drawing on the IPM, we invite a broader interpretation. Thus, part of living well with diabetes involves learning how to relate to emotions with care and flexibility, in ways that acknowledge the mourning of a loss of ease, lost certainty, and the weight of impossible standards.

#### 2.2.1 Ultimate concern – freedom

Diabetes takes away freedom. Put differently, it imposes a form of control that often stands in tension with the desire to live freely. Control is a particularly charged concept for people with diabetes. It points both to what health care professionals advise them to pursue (glycemic control) and to what they often fear (being controlled by the condition). While the language of management is often used to soften this, the lived reality for many remains a continual struggle toward control, or with control itself. This creates an existential bind. To take control of diabetes is to accept a life of constant vigilance, with all the restrictions and emotional effort that this entails. But to reject that vigilance in pursuit of freedom is to risk that diabetes itself takes control of the body in the here and now and, over time, of future health. Either way, control is never neutral (50, 51). It shapes identity, limits spontaneity, and demands continuous negotiation.

Even the modern devices that undoubtedly help many people with diabetes to live well bring with them a kind of noise. Their constant presence, along with the steady stream of data and advice from social media, can quietly chip away at a sense of spontaneity or freedom to just live.

#### 2.2.2 Existential tension - taking responsibility (actio) and undergoing (passio)

In the IPM, the existential tension within the emotional dimension is described as the polarity between taking responsibility (actio) and undergoing (passio), between doing and being (14). This tension reflects the inner conflict of grief and the need to remain open to what is happening, to not flee from pain, and to honour the emotional reality of loss, while also needing to protect oneself and continue living (14). It is not a question of choosing one over the other, but of moving between the two. To grieve well, the model suggests, is to allow both sides of this polarity, to make room for difficult emotions, and to step away from them when needed.

This tension is deeply familiar to many people living with diabetes. A study on diabetes burnout among adults with type 1 diabetes described internal dialogues in which participants struggled between wanting to give up on self-care and feeling that they must not (47). They knew what they were “supposed” to do, yet felt emotionally unable to act, suspended between guilt, exhaustion, and responsibility. Similarly, in a study of binge eating among people with type 2 diabetes, participants described compensatory eating in response to guilt and shame, seeking comfort through food, even as it deepened their distress (32). Both examples speak to the emotional bind that can develop when self-regulation becomes entangled with self-blame.

For many, the daily management of diabetes becomes a site where this existential tension plays out and where the tension between feeling in control or feeling helplessness is a daily struggle (37). The IPM helps us see this not simply as a behavioural challenge or a failure of compliance, but as a deeply human process of emotional negotiation, in which people are doing their best to adapt to loss while continuing to live.

#### 2.2.3 The orientation of emotion regulation, contact with grief and care for self

In the IPM, the orientation in the emotional dimension is described as emotion regulation, that is, the capacity to stay with deep emotion when needed, but also to modulate or soften emotional intensity in order to function and care for oneself. It is not about suppression, but about preserving emotional energy and flexibility.

A central idea here is that the antidote to helplessness is helpfulness. This does not always have to come from others; it can begin by helping oneself, or even by helping others, as a way to restore a sense of agency and meaning.

In diabetes care, feelings of helplessness are common. The emotional burden of managing a relentless, often invisible condition can leave people feeling that their efforts never quite measure up. In this context, emotion regulation becomes more than a therapeutic goal, it is a practical survival skill. Supporting people to identify small, meaningful actions can be a powerful way to rebuild a sense of emotional agency, and with it, the capacity to stay engaged in care (52–54).

### 2.3 IPM & diabetes: the cognitive dimension

The cognitive manifestations of grief can include a sense of disbelief, preoccupation with thoughts of the loss, rumination, counterfactual thinking, problems with concentration and memory. People living with grief continuously interpret their own experiences and strive to find meaning in what happens. This is also called meaning construction (14).

The need to construct a personal understanding of what diabetes means, and to reconstruct one’s view of the self in light of the condition, is well documented in diabetes research (55). Meaning-making in diabetes involves grappling with what has been lost, be it spontaneity, a sense of invulnerability, or imagined futures, and redefining goals and values accordingly. Like grief, this is not a linear process. It may recede into the background at times, only to return with new intensity in response to life transitions, complications, or other disruptions. Understanding this as part of an ongoing learning process, within and alongside grief, can help reframe these fluctuations as natural rather than problematic.

#### 2.3.1 Ultimate concern freedom

In the IPM, the ultimate concern linked to the cognitive dimension is also freedom, more specifically, the freedom to think, imagine, and make meaning without constraint (14). For people living with diabetes, this freedom is compromised. The condition demands constant mental attention: calculating, anticipating, remembering, deciding. The freedom to disregard the body, to act without planning, or to forget the future for a while, is lost.

This loss of cognitive freedom is not just about a practical burden, but about how diabetes inhabits the mind, shaping how people think about themselves, their future, and their safety. Even when glucose levels are stable, the possibility of disruption remains close. The mind must stay alert, even at rest. Unlike other losses, the loss of cognitive ease may be harder to name. It is a quiet, but relentless, demand on the person’s inner life.

#### 2.3.2 Existential tension reflective awareness – embodied awareness

In the IPM, the existential tension in this dimension lies between reflective awareness (the conscious, interpretive understanding of one’s situation) and embodied awareness (the lived, physical experience of being in a vulnerable body) (14). For people with diabetes, this tension is often brought to the surface in moments of bodily unpredictability: a sudden hypoglycemic event during a meeting (56), the soreness from repeated injections (57), or the awkward bulge of a pump or sensor during intimacy (58, 59). These are not only physical interruptions, but moments that jolt a person into awareness of their condition— they are, applying Leder’s portmanteau (60), dysappearance. The challenge lies in holding these two dimensions together: being able to think about diabetes, its implications, and meanings, while still inhabiting the body without fear or detachment. This kind of reflective-embodied integration is rarely discussed in clinical care, yet it may be central to how people live well with diabetes over time.

#### 2.3.3 The orientation of understanding and being aware of grief, honoring and expressing it meaningfully

Orientation in the cognitive dimension involves getting to know one’s grief, becoming aware of how thoughts, beliefs, and bodily signals shape the experience of loss. This reflective process does not remove grief but makes it more navigable. As one becomes more familiar with its contours, the grief becomes less overwhelming and more integrated into daily life. This work often surfaces emotionally charged themes, such as guilt, shame, hope, gratitude, even love. Developing a capacity to move between perspectives, to hold contradictions, and to adjust one’s thinking in response to new realities calls for cognitive flexibility. People with diabetes face not only the practical demands of management but also shifting bodily and emotional landscapes. The ability to notice and reflect on these moments, without becoming stuck in them, is part of what sustains long-term self-care.

Structured therapies such as cognitive behavioural therapy and Acceptance and Commitment Therapy explicitly aim to enhance this kind of cognitive flexibility and have been shown to reduce diabetes distress (61, 62). These approaches are referenced here as examples of existing clinical frameworks that work with meaning-making and perspective-shifting, rather than as comprehensive models of grief. Beyond formal interventions, many people do this work informally, adjusting expectations, shifting goals, and redefining what it means to live well as their condition evolves (63).

### 2.4 IPM & diabetes: the social dimension

In the IPM, the social dimension of grief centers on the deeply relational nature of human life (14). Grief is never purely private; it unfolds within a network of social roles, expectations, and interactions (64). This dimension attends to how people are held, mirrored, or misrecognized by others as they navigate loss. It includes the need to be seen, heard, and acknowledged in one’s grief, as well as the disruptions that occur when those around us are unable or unwilling to bear witness to that grief. Social grief can arise not only from the absence of support but from the strain of maintaining roles, fulfilling obligations, or being treated as unchanged when profound change has occurred. At its heart, this dimension underscores that grieving is a relational process, shaped by the presence, or absence, of meaningful connection.

In the context of diabetes, the social dimension takes on particular import. Living with a chronic condition often requires a continual renegotiation of relationships with family, friends, colleagues, and healthcare professionals. While some people find understanding and support, others encounter misunderstanding, awkwardness, or silence. A recurring challenge lies in how diabetes becomes visible or invisible at different times, and not always in ways that feel safe or manageable. Injecting in public, responding to hypoglycemia, or explaining one’s needs around food or routine can make the condition suddenly, and sometimes uncomfortably, public. At other times, the emotional and physical burden of diabetes remains unseen or dismissed.

These moments of unwanted exposure or social misrecognition can contribute to a deep sense of disconnection. In the workplace, managing diabetes discreetly often demands extra emotional labour, particularly when disclosure carries risks or when support is lacking (65). Even in healthcare settings, where understanding is expected, people may feel reduced to numbers or feel that their emotional experience is overlooked (66). These social dynamics are not incidental. They shape how people experience grief and how they come to terms with the ongoing presence of the condition. The IPM encourages us to recognise these relational contexts and ruptures as central to how people live with loss over time (14, 20).

#### 2.4.1 The ultimate concern of isolation

The ultimate concern in the social dimension is the experience of aloneness and the deep human need for connection. Loss and grief often bring aloneness and anxiety, and for people with diabetes, this can be reflected in feeling different, fearing rejection or being a burden, and worrying about how others perceive them (32). These fears may lead some to withdraw socially, avoiding conversations about diabetes or situations where their condition might be noticed.

At the same time, social connection remains a vital source of strength and support. People with diabetes rely on relationships with family, friends, coworkers, and healthcare professionals, not only for practical assistance but also for emotional understanding and acceptance. While some individuals feel judged or misunderstood within these relationships, others draw significant comfort and resilience from supportive social networks (67, 68).

Navigating this complex social landscape, the need for belonging alongside the fear of exposure or stigma, is central to the lived experience of diabetes. Recognizing and addressing this concern is essential to providing compassionate and effective care.

#### 2.4.2 The existential tension between aloneness and connectedness

The existential tension in the social dimension lies between aloneness and connection. Grief often brings aloneness and anxiety, which in diabetes can manifest as feeling different, fearing being a burden, or losing social ties. This tension between the need for connection and the impulse to retreat is a central human challenge, one that requires not just clinical attention but compassionate understanding.

Existential tension in diabetes is, therefore, deeply social in nature, shaped by how individuals experience belonging, recognition, and difference in relation to others. Social relationships may provide affirmation, continuity, and practical support that help sustain a sense of self in the face of loss. At the same time, they can become sites of strain, misunderstanding, or judgement, where diabetes marks the individual as different or burdensome. Experiences of social stigma, whether subtle or overt, may challenge a person’s sense of legitimacy and worth, undermining not only social participation but also existential security. Importantly, social support and social strain are not opposing categories but often coexist within the same relationships over time. Attending to existential loss in diabetes therefore requires sensitivity to how social contexts can both stabilise and destabilise meaning, shaping how people negotiate identity, autonomy, and their place in the world.

#### 2.4.3 The orientation: continuing bond, seeking support, and letting go

Although aloneness is often feared in the context of diabetes, it is important to recognise that aloneness is not inherently negative. For some, spending time alone can serve as a meaningful form of coping, creating space to reflect, to regain balance, or simply to rest from the social effort of explaining or justifying their condition. This duality reflects the complexity of the social dimension of grief: e.g. while connection can heal, withdrawal can also protect.

The challenge in this dimension is to navigate and balance these needs, maintaining meaningful bonds, seeking support when it is helpful, and recognising when to step back from interactions that consistently undermine or deplete. This balance is not fixed but fluid, shaped by temperament, personal history, and the quality of past relationships. Supporting this kind of relational discernment may be as important as encouraging connection itself.

### 2.5 IPM & diabetes: the spiritual dimension

The spiritual dimension of grief refers to the ways in which people seek meaning, purpose, and connection. This may involve connecting to themselves, to others, to nature, or to something greater (69). Spirituality in this context is not limited to religious belief but includes the search for understanding, coherence, and transcendence. It is the space where questions quietly emerge about why something happened, what matters now, and how to go on.

Spirituality and meaning-making also have a place in the experience of diabetes (70–73), even if they are less often explored than psychological or behavioural responses. Some studies have found that higher levels of reported spirituality are associated with better outcomes, such as lower HbA1c in young adults (71). Yet the deeper role of spirituality in shaping how people live with diabetes remains less well understood. In one study of individuals with type 1 diabetes, the early period following diagnosis was described as a moment of profound change. It altered not only how people saw themselves but also how they related to the condition, to others, and to the healthcare system (74). For some, diabetes became a context for making sense of life in new ways. For others, it remained a burdensome medical task.

These responses are not fixed. They are part of an ongoing process of adaptation. Just as grief can lead people to revisit their identity or beliefs, so too can chronic illness prompt reflection on what matters, where strength is found, and how meaning is made.

#### 2.5.1 Ultimate concern transcendence

Transcendence is one of the more elusive but meaningful dimensions of grief. In the context of chronic illness, it does not refer to spiritual elevation or escape but to the capacity to move beyond immediate physical and emotional difficulty and reconnect with life in a fuller, more integrated way. In the IPM, transcendence involves engaging with what lies outside our control and sometimes even beyond words. This may include trust, faith, and a reawakening of purpose.

When a person is confronted with loss, whether of health, certainty, or a once-imagined future, this sense of orientation can collapse. The spiritual question becomes whether one can believe in life again. This is not necessarily about religion but about the existential challenge of rebuilding a life that feels livable.

In one study, participants with type 1 diabetes described how emotional upheavals such as stress, trauma, or grief could make their condition feel unpredictable and unresponsive. One participant explained that their diabetes would behave in ways that were “beyond reason” and “difficult to explain” (51) such moments, the condition becomes a symbol of what cannot be controlled, what shifts without warning, and what must still be lived with. For some, this unpredictability invites reflection and reevaluation about what matters, what can be trusted, why did it happen to me, and how to keep living meaningfully even when certainty is gone.

#### 2.5.2 Existential tension: meaning and meaninglessness

The human mind naturally seeks meaning in the face of adversity. In the IPM, the existential tension between meaning and meaninglessness is described as a continuum between knowing and believing (14). This tension plays out as individuals navigate the limits of certainty, science, and reason alongside the need for personal belief, hope, or trust in something beyond what can be measured.

In the context of diabetes, this tension often appears in the struggle to understand and manage a condition that is sometimes highly rational and at other times deeply unpredictable. Qualitative studies of people living with diabetes frequently describe a longing for ‘normality’ e.g. a life unmarked by medical routines, blood sugar fluctuations, and constant self-surveillance. Parents of children with diabetes, adolescents, and adults alike often strive toward this goal even as they recognize it may be unattainable (30, 75).

Living with diabetes involves holding two truths at once: the need to act based on knowledge, such as carb counting, insulin doses, and glycaemic targets, and the parallel need to sustain belief in a meaningful life even when things feel out of control or overwhelming. This inner balancing act reflects the broader human challenge of finding coherence in a world that does not always behave as expected (76).

#### 2.5.3 Orientation being open to resonance finding new meaningful goals

In the IPM, the orientation of the spiritual dimension is described as the search for new meaningful goals (14). This involves reconnecting with values, forging new pathways, and reengaging with life in a way that resonates with the person’s changed sense of self. This process does not follow a linear path. It unfolds in ways shaped by individual histories, relationships, and the cumulative experience of loss. The search for meaning is not separate from the other dimensions in the model. It draws on them and contributes to them, weaving together emotional, physical, cognitive, social, and spiritual experience.

In the context of diabetes, this orientation often emerges gradually. While diagnosis may initially feel like a rupture, many people begin to reweave it into their sense of self (77). Some find purpose through advocacy, others deepen their care for loved ones, and some quietly shift their understanding of what it means to live well with limitations. For many, diabetes becomes part of how they make sense of the world. It is not a chosen path, but it can become one that invites reflection, strength, and at times growth. Even in the presence of distress, diabetes can be integrated into a person’s life story in a way that holds rather than fragment identity. This is not about making diabetes meaningful, but about finding meaning in the life that continues alongside it.

## 3 Discussion

### 3.1 Applying the integrative process model in practice: lessons from patient partnership

The practical value of the Integrative Process Model lies in how its dimensions can be translated into real-world care and engagement. In diabetes research and system design, grief-literate practice involves creating environments where loss, adaptation, and meaning-making are recognised as integral to participation.

One example of this translation can be seen in patient-partnership initiatives within Diabetes Action Canada, where people with lived and loved experience of diabetes co-design research, policy, and educational tools (78, 79). Drawing from both the IPM framework and trauma-informed principles, these initiatives integrate the physical, emotional, cognitive, social, and spiritual dimensions of grief into daily collaboration.

Many of these grief-literate methods echo findings from earlier co-design work (78), which identified symbolic markers, patient-led agenda setting, and intentional rituals of reflection as mechanisms for fostering belonging, dignity, and balance in collaborative environments. Examples include beginning sessions with non-clinical icebreakers to build connection, sharing clear agendas and pre-reads to reduce anxiety, offering participants the option to pause or step back, providing equitable compensation that acknowledges emotional labour, co-facilitating meetings to balance power, and closing with reflection rather than abrupt endings. Emotional expressions are treated as information about what matters most, not as disruptions.

Together, these approaches form a grief-literate method of engagement that honours vulnerability as a source of insight and reciprocity on a path toward integration. They illustrate how the five dimensions of the IPM can guide concrete, sustainable methods for partnership and care. **Table 2** summarises these practices and demonstrates how grief literacy can be operationalised within patient-oriented research contexts.

**Table 2:** Translating the integrative process model into practice: examples of grief-literate approaches to patient partnership.

IPM dimension	Grief-literate practice	Illustrative example in implementation
Physical	Create predictability and comfort to reduce physiological stress.	Share agendas and pre-reads in advance; include clear time boundaries; offer the option to step out or turn off camera; open sessions with light, non-clinical icebreakers (e.g., “favourite cereal”, “tiny recent joy”) to humanize connection and ease entry.
Emotional	Recognize and regulate affect as part of participation.	Build pause points or check-ins during heavy topics; model emotional naming; treat moments of silence or tears as valid forms of contribution.
Cognitive	Support sense-making and integration of experience.	Invite participants to share “what felt important” alongside “what we learned”; use lay language summaries; co-create visual or creative outputs that transform insight into shared knowledge.
Social	Foster safety, reciprocity, and equity within power dynamics.	Co-chair sessions with patient partners; rotate speaking order; ensure compensation parity and transparency; use relational rituals such as shared reflections to close each meeting.
Spiritual	Enable contribution and meaning-making as paths to purpose.	Design projects that allow lived experience to inform change (e.g., co-developing tools, creative outputs, or mentorship roles); name and honour contributions publicly.
Across dimensions	Embed care and repair into the structure of engagement.	End each cycle with debriefs or “share-backs”; follow up within 24–48 h after intense sessions; close meetings with intentional warmth (e.g., “no creepy goodbyes”—everyone unmutes, waves, or shares one word); document process learnings to strengthen future collaborations.

Examples draw on practices developed within Diabetes Action Canada’s national patient partnership program (45), illustrating how the five dimensions of the Integrative Process Model can inform design and facilitation in chronic illness contexts.

The practical strategies outlined above demonstrate how the IPM can be embodied in everyday patient–partner encounters, grounding abstract principles of loss and grief in the small gestures and relational rituals of care. Yet, while these practices make the model tangible, their power lies in what they reflect conceptually: that adaptation to life with diabetes unfolds across multiple, interwoven dimensions—physical, emotional, cognitive, social, and spiritual. At present, there are few models in diabetes care that explicitly frame emotional life through the lens of loss and grief. This is why we draw on Diabetes Action Canada as an illustrative, care-adjacent example rather than a clinical exemplar. Its relevance lies in showing how processes central to the Integrative Process Model, such as acknowledgment of loss, adaptation, and meaning-making, can be supported in practice, even outside formal clinical encounters.

A grief-informed perspective does not require new clinical models so much as a reorientation of existing ones. Within routine care, attention could be given not only to distress or burden, but also to cumulative and ambiguous losses over time, and clinicians could be supported to acknowledge these experiences without feeling compelled to resolve them. In this way, principles from the Integrative Process Model could be integrated into everyday diabetes care alongside established psychosocial approaches.

### 3.2 IMP as a model for diabetes care - viewing the consequences of diabetes through the lens of grief

In this paper, we have argued that while the construct of diabetes distress has brought essential clarity and legitimacy to the emotional burdens of diabetes, it does not fully capture the more diffuse, cumulative, and existential changes that many people describe across the long course of illness. The Integrative Process Model offers a way to hold these quieter dimensions in view. Its value lies not in redefining distress but in situating it within a broader and more textured landscape of adaptation that includes loss of spontaneity, shifts in identity, altered bodily trust, and the steady recalibration of meaning. Bringing these dimensions together makes visible aspects of emotional life that often remain unnoticed in routine care and underexplored in research.

Each dimension of the IPM reflects a fundamental existential tension that shapes how people navigate life with diabetes. In the physical dimension, the tension lies between embracing life and recognising the limitations imposed by illness. The body becomes both a site of vitality and a reminder of vulnerability, demanding vigilance and care. In the emotional dimension, the tension unfolds between undergoing and acting, between allowing emotional responses to surface and managing them in order to sustain everyday life. The cognitive dimension reveals the tension between reflective awareness and embodied experience, as people work to make sense of their condition while living with its unpredictable physical realities. The social dimension centers on the pull between aloneness and connection, highlighting how diabetes can both disrupt and deepen relationships. And in the spiritual dimension, the existential tension lies between meaning and meaninglessness and the challenge of sustaining belief, hope, or trust in the face of chronic uncertainty.

Taken together, these five dimensions allow us to view life with diabetes not only as a matter of blood glucose management or psychological resilience, but as a complex, ongoing process of adaptation to life in the face of loss. The language of loss and grief helps frame these experiences, not as symptoms or signs of dysfunction, but as deeply human responses to change.

At the same time, we recognise that applying an integrative model developed in the context of loss and grief to chronic illness requires careful reflection. In older grief models, grief is often seen as a response to a specific loss, with physical and emotional symptoms that fade or resolve over time. In diabetes, the losses are ongoing, cumulative, and continually on the horizon. The body is not simply a site of temporary reaction but remains a central, persistent focus of attention, concern, and sometimes frustration. People with diabetes do not experience loss and then move on. Rather, they live within a shifting landscape of loss and adaptation, where new routines must be negotiated again and again. Some aspects of the IPM, particularly the physical dimension, may therefore require adaptation and refinement to better reflect the nature of chronic conditions. The concept of reactivation, for instance, may look different when the trigger is not a memory or anniversary, but a hypoglycemic episode, a failed injection, a disrupted meal, or a moment of unexpected public visibility. Reactions are not reawakened from the past but called forth in real time, often without pause.

Another way the perspective of loss and grief can be extended in diabetes care is by recognising that these experiences are not only personal but also shaped by broader social and cultural forces. In type 2 diabetes especially, the condition is often entangled with structural factors such as economic precarity, food insecurity, limited healthcare access, racialised stigma, and cumulative social exclusion (80–82). In many communities, diabetes is not just a medical diagnosis, but the visible consequence of systemic loss. This recognition invites a broader understanding of adaptation and meaning making, one that takes into account not only individual responses but also the external conditions that shape the possibility of living well. Nowhere is this more urgent than in communities that have endured collective loss, such as Indigenous populations, where the burden of diabetes cannot be separated from histories of dispossession and discrimination. Here, grief is not only emotional but also cultural and political, and its presence is written into the aetiology of chronic illness itself.

Of course, this does not mean that people with type 1 diabetes are untouched by such forces. Indeed, many also encounter stigma, misunderstanding, and systemic failure (83). What it highlights, rather, is the need to understand diabetes not simply as a biological diagnosis, but as a condition that interacts with broader structures of meaning, identity, and power.

By viewing the consequences of diabetes through the lens of loss and grief, we open space for a richer emotional vocabulary in both research and care. We also bring into view the many ways people resist, adapt to, and live meaningfully with chronic illness. Grief, as understood in the IPM, is not a sign of weakness or something to be worked through and left behind. It is part of what it means to be human in the face of change. For people living with diabetes, acknowledging this may help make room for not only distress, but for reflection, connection, and growth.

### 3.3 Implications for practice

Ultimately, the purpose of introducing the perspective of loss and grief into the encounter between healthcare professionals and people living with diabetes is to normalize conversations about the strain and transformation that often accompany chronic illness. Here, the task of the healthcare professional is not to remove the burden or the grief, but rather to offer support so that the person, or family, affected by diabetes can find or reestablish a sense of balance.

From clinical work with children and adolescents living with diabetes in Denmark, we know there is an effort to frame the condition in a way that emphasizes that the child is not ill, but rather undergoing treatment to prevent future illness. While this can reduce unnecessary fear, it can also create a reticence, shared between parents, clinicians, and sometimes the child, about what is at stake. The possibility of complications, loss of control, even death, may linger in the background, unspoken. Introducing grief as a natural, human response to chronic uncertainty could open space for a more nuanced dialogue where it is legitimate to articulate a sense of vulnerability without necessarily having to relate to it as something which needs to be resolved. Similarly, in encounters with adults living with diabetes, the perspective of loss and grief could provide a language for expressing the burden they carry. It would allow for an open conversation about strain and distress without framing these experiences as symptoms of illness.

In this way, the IPM is also a perspective that aligns with the literature on diabetes distress, which consistently emphasises that distress is not a psychological disorder but a normal response to the demands of life with diabetes. Introducing the IPM into diabetes care offers a gentle but clear structure for recognising and responding to these experiences. It gives clinicians a way to talk about emotional fluctuations, rigid thoughts, sensations of aloneness, or protective withdrawal, not as signs of failure but as understandable human responses. It supports conversations about emotional regulation and cognitive effort without assuming these efforts reflect something wrong. Most of all, it invites a deeper encounter with the person behind the condition, grounded in insight and respect.

This approach may also have added benefits for healthcare professionals. Many feel a sense of helplessness when confronted with what cannot be fixed (84). Loss and grief, introduced as a shared experience rather than a clinical problem, can restore a sense of purpose. It can renew a connection to the human core of care. Naming what is difficult can make it more bearable, not only for the person living with diabetes but also for the people walking alongside them.

Although the model does not immediately translate on its own into practical recommendations, the IPM can become clinically useful with further work to develop practical tools, training, and reflective frameworks to guide healthcare professionals in how to listen, respond, and create space for these experiences. What we offer here is a conceptual starting point. Its full value depends on how it is taken up, adapted, and applied in real-world care. This is not a call for more emotional labour, but an invitation to recognise what is already present in clinical encounters and to offer language, structure, and support for engaging with it in ways that feel both sustainable and meaningful.

## 4 Conclusion

In this paper, we have introduced grief as a conceptual lens for understanding the human experience of living with diabetes. Drawing on literature across the life course, we have argued that naming and articulating loss gives shape to what is often felt but rarely spoken. While the construct of diabetes distress has brought welcome attention to the emotional toll of life with diabetes, it may not fully account for the subtler and more existential forms of suffering that can emerge over time.

By turning to the IPM, we place distress within a broader emotional and social landscape. This includes mourning for lost spontaneity, certainty, bodily trust, or imagined futures. Grief helps us recognise that some forms of distress are not problems to solve but truths to honour. In doing so, we do not replace the concept of distress but deepen it. This perspective offers a compassionate and contextually grounded understanding of what people live with and what they live without.

Introducing this language into diabetes care may help normalise human suffering, reduce stigma, and guide healthcare professionals in offering support that is not only clinically sound but emotionally attuned. It invites us to listen in a different way, not just for symptoms but for meaning.

## References

[1] Wiebe DJ Helgeson V Berg CA . The social context of managing diabetes across the life span. Am Psychol. (2016) 71:526–38. doi: 10.1037/a0040355. PMID: 27690482 PMC5094275

[2] Davies M . Psychological aspects of diabetes management. Medicine. (2022) 50:749–51. doi: 10.1016/j.mpmed.2022.08.011. PMID: 38826717

[3] Polonsky WH Anderson BJ Lohrer PA Welch G Jacobsen AM Aponte JE . Assessment of diabetes-related distress. Diabetes Care. (1995) 18:754–760. doi: 10.2337/diacare.18.6.754, PMID: 7555499

[4] Fisher L Glasgow RE Mullan JT Skaff MM Polonsky WH . Development of a brief diabetes distress screening instrument. Ann Family Med. (2008) 6:246–252. doi: 10.1370/afm.842, PMID: 18474888 PMC2384991

[5] Fisher L Polonsky WH Hessler DM Masharani U Blumer I Peters AL . Understanding the sources of diabetes distress in adults with type 1 diabetes. J Diabetes Its Complications. (2015) 29(4):572–7. doi: 10.1016/j.jdiacomp.2015.01.012. PMID: 25765489 PMC4414881

[6] Fisher L Gonzalez JS Polonsky WH . The confusing tale of depression and distress in patients with diabetes: a call for greater clarity and precision. Diabetic Med. (2014) 31:764–72. doi: 10.1111/dme.12428. PMID: 24606397 PMC4065190

[7] Nouwen A . Depression and diabetes distress. Diabetic Med. (2015) 32:1261–1263. doi: 10.1111/dme.12863, PMID: 26202578

[8] Dieter T Lauerer J . Depression or diabetes distress? Perspect Psychiatr Care. (2018) 54:84–7. doi: 10.1111/ppc.12203. PMID: 28090642

[9] Skinner TC Joensen L Parkin T . Twenty-five years of diabetes distress research. Diabetic Med. (2020) 37:393–400. doi: 10.1111/dme.14157. PMID: 31638279

[10] Hendrieckx C Halliday JA Beeney LJ Speight J . (2021). Diabetes and emotional health: A practical guide for health professionals supporting adults with type 1 and type 2 diabetes. Arlington: American Diabetes Association. 10.2196/15007 PMC7060499 32130112

[11] Young-Hyman D de Groot M Hill-Briggs F Gonzalez JS Hood K Peyrot M . Psychosocial care for people with diabetes: A position statement of the American Diabetes Association, New York. Diabetes Care. (2016) 39:2126–2140. doi: 10.2337/dc16-2053, PMID: 27879358 PMC5127231

[12] Due-Christensen M Willaing I Ismail K Forbes A . Learning about type 1 diabetes and learning to live with it when diagnosed in adulthood: two distinct but inter-related psychological processes of adaptation a qualitative longitudinal study. Diabetic Med. (2019) 36(6):742–52. doi: 10.1111/dme.13838, PMID: 30329176

[13] Stenov V Due-Christensen M Christensen JN Willaing I Cleal B . Discovering the hidden emotional burden: Systematic screening for diabetes distress in adults with type 1 diabetes in nurse-led routine diabetes care. Diabetic Med. (2025) 42:e70064. doi: 10.1111/dme.70064, PMID: 40373169 PMC12151820

[14] Guldin M-B Leget C . Loss, grief and existential awareness: An integrative approach. London: Routledge (2024). doi: 10.4324/9781003499060

[15] Snoek FJ Bremmer MA Hermanns N . Constructs of depression and distress in diabetes: Time for an appraisal. Lancet Diabetes Endocrinol. (2015) 3:450–460. doi: 10.1016/S2213-8587(15)00135-7, PMID: 25995123

[16] Fisher L Polonsky WH Hessler D . Addressing diabetes distress in clinical care: a practical guide. Diabetic Med. (2019) 36:803–812. doi: 10.1111/dme.13967, PMID: 30985025

[17] Fisher L Guzman S Polonsky W Hessler D . Bringing the assessment and treatment of diabetes distress into the real world of clinical care: Time for a shift in perspective. Diabetic Med. (2024) 41:e15446. doi: 10.1111/dme.15446, PMID: 39393003

[18] Fisher L Mullan JT Arean P Glasgow RE Hessler D Masharani U . Diabetes distress but not clinical depression or depressive symptoms is associated with glycemic control in both cross-sectional and longitudinal analyses. Diabetes Care. (2010) 33:23–28. doi: 10.2337/dc09-1238, PMID: 19837786 PMC2797978

[19] Charmaz K . Loss of self: a fundamental form of suffering in the chronically ill. Sociology Health Illness. (1983) 5:168–195. doi: 10.1111/1467-9566.ep10491512, PMID: 10261981

[20] Guldin M-B Leget C . The integrated process model of loss and grief - An interprofessional understanding. Death Stud. (2024) 48:738–752. doi: 10.1080/07481187.2023.2272960, PMID: 37883693

[21] Yalom I . (1980). Existential psychotherapy. New York: Basic Books.

[22] Fagundes CP Brown RL Chen MA Murdock KW Saucedo L LeRoy A . Grief, depressive symptoms, and inflammation in the spousally bereaved. Psychoneuroendocrinology. (2019) 100:190–197. doi: 10.1016/j.psyneuen.2018.10.006, PMID: 30368120 PMC6889080

[23] Brown RL LeRoy AS Chen MA Suchting R Jaremka LM Liu J . Grief symptoms promote inflammation during acute stress among bereaved spouses. Psychol Sci. (2022) 33:859–873. doi: 10.1177/09567976211059502, PMID: 35675903 PMC9343888

[24] Nirupama R Rajaraman B Yajurvedi HN . Stress and Glucose metabolism: A review. Imaging J Clin Medical Sci. (2018) 5(1): 008–012.

[25] Déniz-García A Díaz-Artiles A Saavedra P Alvarado-Martel D Wägner AM Boronat M . Impact of anxiety, depression and disease-related distress on long-term glycaemic variability among subjects with type 1 diabetes mellitus. BMC Endocr Disord. (2022) 22:122. doi: 10.1186/s12902-022-01013-7, PMID: 35546667 PMC9092877

[26] Colton P Rodin G Bergenstal R Parkin C . Eating disorders and diabetes: Introduction and overview. Diabetes Spectr. (2009) 22:138–42. doi: 10.2337/diaspect.22.3.138

[27] Gal AM Iatcu CO Popa AD Arhire LI Mihalache L Gherasim A . Understanding the interplay of dietary intake and eating behavior in type 2 diabetes. Nutrients. (2024) 16:771. doi: 10.3390/nu16060771, PMID: 38542683 PMC10975878

[28] Powers SW Byars KC Mitchell MJ Patton SR Standiford DA Dolan LM . Parent report of mealtime behavior and parenting stress in young children with type 1 diabetes and in healthy control subjects. Diabetes Care. (2002) 25:313–318. doi: 10.2337/diacare.25.2.313, PMID: 11815502

[29] Young V Anderson BJ Lohrer PA Welch G Jacobsen AM Aponte JE . Eating problems in adolescents with type 1 diabetes: A systematic review with meta-analysis. Diabetic Med. (2013) 30:189–198. doi: 10.1111/j.1464-5491.2012.03771.x, PMID: 22913589

[30] Nielsen BK Jensen AL Ryberg A Pedersen SB Lund S Lou S . It's a balancing act. A qualitative study of the everyday management of type 1 diabetes among people with unexplained persistent hyperglycaemia. Scandinavian J Caring Sci. (2025) 39:e70001. doi: 10.1111/scs.70001, PMID: 40040551 PMC11880960

[31] Chevinsky JD Wadden TA Chao AM . Binge eating disorder in patients with type 2 diabetes: Diagnostic and management challenges. Diabetes Metab Syndrome Obes. (2020) 13:1117–1131. doi: 10.2147/DMSO.S213379, PMID: 32341661 PMC7166070

[32] Lindgreen P Willaing I Clausen L Ismail K Grønbæk HN Andersen CH . 'I haven't told anyone but you': Experiences and biopsychosocial support needs of people with type 2 diabetes and binge eating. Qual Health Res. (2024) 34(7):621–634. doi: 10.1177/10497323231223367, PMID: 38183221 PMC11103901

[33] Huffhines L Noser A Patton SR . The link between adverse childhood experiences and diabetes. Curr Diabetes Rep. (2016) 16:54. doi: 10.1007/s11892-016-0740-8, PMID: 27112958 PMC5292871

[34] Herbert LJ Monaghan M Cogen F Streisand R . The impact of parents' sleep quality and hypoglycemia worry on diabetes self-efficacy. Behav Sleep Med. (2015) 13:308–323. doi: 10.1080/15402002.2014.898303, PMID: 24738994 PMC4199924

[35] Tracy EL Berg CA Baucom KJW Turner SL Kelly CS Van Vleet M . Daily sleep quality and daily stressors in couples coping with type 1 diabetes. Health Psychol. (2019) 38:75–83. doi: 10.1037/hea0000690, PMID: 30372105 PMC6309199

[36] Macaulay GC Boucher SE Yogarajah A Galland BC Wheeler BJ . Sleep and night-time caregiving in parents of children and adolescents with type 1 diabetes mellitus – A qualitative study. Behav Sleep Med. (2020) 18:622–636. doi: 10.1080/15402002.2019.1647207, PMID: 31370700

[37] Morales-Brown LA Perez Algorta G Salifu Y . Understanding experiences of diabetes distress: A systematic review and thematic synthesis. J Diabetes Res. (2024) 2024:3946553. doi: 10.1155/2024/3946553, PMID: 39574786 PMC11581805

[38] Streisand R Swift E Wickmark T Chen R Holmes CS . Pediatric parenting stress among parents of children with type 1 diabetes: The role of self-efficacy, responsibility, and fear. J Pediatr Psychol. (2005) 30:513–521. doi: 10.1093/jpepsy/jsi076, PMID: 16055489

[39] Peter ME Rioles N Liu J Chapman K Wolf WA Nguyen H . Prevalence of fear of hypoglycemia in adults with type 1 diabetes using a newly developed screener and clinician's perspective on its implementation. BMJ Open Diabetes Res Care. (2023) 11:e003394. doi: 10.1136/bmjdrc-2023-003394, PMID: 37423638 PMC10335552

[40] Emmanuel Sochukwuma E Nassif RN Chibuike OP Okeke S Chukwubuzo OT Ekpunobi CP . Relationship between death anxiety and health related quality of life among diabetic patients: The predictive roles of experiential avoidance. Global J Obesity Diabetes Metab Syndrome. (2022) 9:11–19. doi: 10.17352/2455-8583.000056

[41] Enea V Candel OS Zancu SA Maftei A Bîrlădeanu L Timofte D . Death obsession, COVID-19-related fear and religiosity in people living with type 2 diabetes. OMEGA - J Death Dying. (2024) 89:1094–1112. doi: 10.1177/00302228221085402, PMID: 35441558 PMC9023313

[42] Farthing P Bally J Rennie DC . Perceptions related to death in adolescents and their parents during the management of type 1 diabetes: A thematic analysis. J Pediatr Health Care. (2024) 38:586–594. doi: 10.1016/j.pedhc.2024.04.002, PMID: 38661590

[43] Chan JCN Lim LL Wareham NJ Shaw JE Orchard TJ Zhang P . The Lancet Commission on diabetes: using data to transform diabetes care and patient lives. Lancet. (2020) 396:2019–2082. doi: 10.1016/S0140-6736(20)32374-6, PMID: 33189186

[44] Carrier MA Beverly EA . Focus on the positive: A qualitative study of positive experiences living with type 1 or type 2 diabetes. Clin Diabetes. (2021) 39:176–187. doi: 10.2337/cd20-0082, PMID: 33981131 PMC8061556

[45] Hortensius J Kars MC Wierenga WS Kleefstra N Bilo HJ van der Bijl JJ . Perspectives of patients with type 1 or insulin-treated type 2 diabetes on self-monitoring of blood glucose: a qualitative study. BMC Public Health. (2012) 12:167. doi: 10.1186/1471-2458-12-167, PMID: 22397638 PMC3311150

[46] Tanenbaum ML Commissariat PV . Experience with burdens of diabetes device use that affect uptake and optimal use in people with type 1 diabetes. Endocrine Connections. (2023) 12:e230193. doi: 10.1530/EC-23-0193, PMID: 37522857 PMC10503226

[47] Abdioli S Hessler D Smither B Miller-Bains K Burr EM Stuckey HL . New insights into diabetes burnout and its distinction from diabetes distress and depressive symptoms: A qualitative study. Diabetes Res Clin Pract. (2020) 169:108446. doi: 10.1016/j.diabres.2020.108446, PMID: 32946853

[48] Inagaki S Matsuda T Muramae N Abe K Kato K . Diabetes-related shame among people with type 2 diabetes: an internet-based cross-sectional study. BMJ Open Diabetes Res Care. (2022) 10:e003001. doi: 10.1136/bmjdrc-2022-003001, PMID: 36593661 PMC9748962

[49] Fisher L Hessler D Polonsky W Stryker L Guzman S Bowyer V . Emotion regulation contributes to the development of diabetes distress among adults with type 1 diabetes. Patient Educ Couns. (2018) 101:124–131. doi: 10.1016/j.pec.2017.06.036, PMID: 28739179 PMC5732076

[50] Mol A Law J . Embodied action, enacted bodies: The example of hypoglycaemia. Body Soc. (2004) 10:43–62. doi: 10.1177/1357034X04042932

[51] Mol A . (2008). The logic of care: health and the problem of patient choice. London: Routledge.

[52] Coccaro EF Lazarus S Joseph J Wyne K Drossos T Phillipson L . Emotional regulation and diabetes distress in adults with type 1 and type 2 diabetes. Diabetes Care. (2020) 44:20–25. doi: 10.2337/dc20-1059, PMID: 33444157 PMC8742145

[53] Kollin SR Gratz KL Lee AA . The role of emotion dysregulation in selfmanagement behaviors among adults with type 2 diabetes. J Behav Med. (2024) 47:672–681. doi: 10.1007/s10865-024-00483-5, PMID: 38671288 PMC11291593

[54] FisherL GuzmanS PolonskyWH StryckerL GreenbergK HesslerDM . How does addressing diabetes distress lead to positive glycemic change? Results from the EMBARK trial. Patient Educ Couns. (2025) 144:108748. doi: 10.1016/j.pec.2025.108748, PMID: 40555621

[55] Due-Christensen Zoffmann V Willaing I Hopkins D Forbes AM . The process of adaptation following a new diagnosis of type 1 diabetes in adulthood: A meta-synthesis. Qual Health Res. (2018) 28:245–258. doi: 10.1177/1049732317745100, PMID: 29235942

[56] Stuckey HL Desai U King SB Popadic L Levinson W Kirson NY . The experience of a severe hypoglycaemic event from the perspective of people with diabetes and their caregivers: 'What am I going to do?'. Diabetic Med. (2022) 39:e14745. doi: 10.1111/dme.14745, PMID: 34797937 PMC9299593

[57] Hanberger L Tallqvist E Richert A Olinder AL Forsner M Morelius E . Needle-related pain, affective reactions, fear, and emotional coping in children and adolescents with type 1 diabetes: A cross-sectional study. Pain Manag Nurs. (2021) 22:516–521. doi: 10.1016/j.pmn.2021.01.007, PMID: 33640255

[58] Messina R Due-Christensen M Keller-Senn A Polek E Fantini MP Sturt J . Couples living with type 1 diabetes: An integrative review of the impacts on health and wellbeing. J Health Psychol. (2021) 26:412–437. doi: 10.1177/1359105318817356, PMID: 30574793

[59] Natale P Chen S Chow CK Cheung NW Martinez-Martin D Caillaud C . Patient experiences of continuous glucose monitoring and sensor augmented insulin pump therapy for diabetes: A systematic review of qualitative studies. J Diabetes. (2023) 15:1048–1069. doi: 10.1111/1753-0407.13454, PMID: 37551735 PMC10755613

[60] Leder D . (1995). The absent body. Chicago: University of Chicago Press.

[61] Schmidt CB van Loon BJP Vergouwen ACM Snoek FJ Honig A . Systematic review and meta-analysis of psychological interventions in people with diabetes and elevated diabetes-distress. Diabetic Med. (2018). doi: 10.1111/dme.13709, PMID: 29896760

[62] Jenkinson E Knoop I Hudson JL Moss-Morris R Hackett RA . The effectiveness of cognitive behavioural therapy and third wave cognitive behavioural interventions on diabetes-related distress: A systematic review and meta-analysis. Diabetic Med. (2022) 39:e14948. doi: 10.1111/dme.14948, PMID: 36031793 PMC9826380

[63] Hessler DM Fisher L Guzman S Stryker L Polonsky WH Ahmann A . EMBARK: A randomized, controlled trial comparing three approaches to reducing diabetes distress and improving HbA1c in adults with type 1 diabetes. Diabetes Care. (2024) 47:1370–1378. doi: 10.2337/dc23-2452, PMID: 38809903 PMC11272976

[64] Sköld A . A social ontology of grief. Theory Psychol. (2023) 33:24–41. doi: 10.1177/09593543221128231

[65] Hansen UM Olesen K Browne JL Skinner TC Willaing I . A call for inclusion of work-related diabetes distress in the spectrum of diabetes management: Results from a cross-sectional survey among working people with type 1 diabetes. Diabetes Res Clin Pract. (2018) 140:139–147. doi: 10.1016/j.diabres.2018.03.040, PMID: 29604390

[66] Litterbach E Holmes-Truscott E Pouwer F Speight J Hendrieckx C . I wish my health professionals understood that it's not just all about your HbA1c!. Qualitative responses from the second Diabetes MILES – Australia (MILES-2) study. Diabetic Med. (2020) 37:971–981. doi: 10.1111/dme.14199, PMID: 31802530

[67] Carrier MA Beverly EA. Focus on the positive: A qualitative study of positive experiencesliving with type 1 or type 2 diabetes. Clin Diabetes. (2021) 39:176–187. doi: 10.2337/cd20-0082, PMID: 33981131 PMC8061556

[68] Gopalan A Blatchins MA Xu KK Altschuler A Marshall CJ Hessler DM . All in the family: A qualitative study of the early experiences of adults with younger onset type 2 diabetes. J Am Board Family Med. (2022) 35:341–351. doi: 10.3122/jabfm.2022.02.210223, PMID: 35379721 PMC9605685

[69] Nolan S Saltmarsh P Leget C . Spiritual care in palliative care: Working towards an EAPC Task Force. Eur Public Law. (2011) 18.

[70] Samuel-Hodge CD Headen SW Skelly AH Ingram AF Keyserling TC Jackson EJ . Influences on day-to-day self-management of type 2 diabetes among African-American women: Spirituality, the multi-caregiver role, and other social context factors. Diabetes Care. (2000) 23:928–933. doi: 10.2337/diacare.23.7.928, PMID: 10895842

[71] Parsian N Dunning T . Spirituality and coping in young adults with diabetes: A cross-sectional study. Eur Diabetes Nurs. (2009) 6:100–104. doi: 10.1002/edn.144, PMID: 41531421

[72] Sridhar GR . Diabetes, religion and spirituality. Int J Diabetes Developing Countries. (2013) 33:5–7. doi: 10.1007/s13410-012-0097-8, PMID: 30311153

[73] Darvyri P Christodoulakis S Galanakis M Avgoustidis AG Thanopoulou A Chrousos GP . On the role of spirituality and religiosity in type 2 diabetes mellitus management—A systematic review. Psychology. (2018) 9:728–744. doi: 10.4236/psych.2018.94046

[74] Paterson B Thorne S Crawford J Tarko M . Living with Diabetes as a Transformational Experience. Qualitative Health Research. (1999) 9:786–802. doi: 10.1177/104973299129122289, PMID: 10662259

[75] Bekker CI Deacon E Segal D . Meaning in life experienced by parents of children living with diabetes. Health Psychol Open. (2019) 6:2055102919832221. doi: 10.1177/2055102919832221, PMID: 30858981 PMC6402055

[76] Zoffmann V Kirkevold M . Life versus disease in difficult diabetes care: Conflicting perspectives disempower patients and professionals in problem solving. Qual Health Res. (2005) 15:750–765. doi: 10.1177/1049732304273888, PMID: 15961873

[77] Raymaekers K Prikken S Vanhalst J Moons P Goossens E Oris L . The social context and illness identity in youth with type 1 diabetes: A three-wave longitudinal study. J Youth Adolescence. (2020) 49:449–466. doi: 10.1007/s10964-019-01180-2, PMID: 31853683

[78] McQuire T Boisvenue J Mackett J Maghera J Makarski J Martin A . Partnering for impact: Best practices for planning in-person academic events with Patient Partners involvement- Lessons learned from Diabetes Action Canada. Res Involvement Engagement. (2025) 11:55. doi: 10.1186/s40900-025-00729-9, PMID: 40420247 PMC12105301

[79] Mytkolli L Ahmed A Ajmal S Pagola AA Binko J Chinchilla P . Diabetes in four dialects: A global call for equity across lived, loved, learned and laboured experiences. Diabetologia. (2025) 68:2308–2317. doi: 10.1007/s00125-025-06506-3, PMID: 40781567

[80] Browne JL Ventura A Mosely K Speight J . 'I call it the blame and shame disease': a qualitative study about perceptions of social stigma surrounding type 2 diabetes. BMJ Open. (2013) 3:e003384. doi: 10.1136/bmjopen-2013-003384, PMID: 24247325 PMC3840338

[81] Hill-Briggs F Adler NE Berkowitz SA Chin MH Gary-Webb TL Navas-Acien A . Social determinants of health and diabetes: A scientific review. Diabetes Care. (2021) 44:258–279. doi: 10.2337/dci20-0053, PMID: 33139407 PMC7783927

[82] Levi R Bleich SN Seligman HK . Food insecurity and diabetes: Overview of intersections and potential dual solutions. Diabetes Care. (2023) 46:1599–1608. doi: 10.2337/dci23-0002, PMID: 37354336 PMC10465985

[83] Liu NF Brown AS Folias AE Younge MF Guzman SJ Close KL . Stigma in people with type 1 or type 2 diabetes. Clin Diabetes. (2017) 35(1):27–34. doi: 10.2337/cd16-0020, PMID: 28144043 PMC5241772

[84] Craven M Simons Z de Groot M . Diabetes distress among healthcare providers: A qualitative study. Diabetes Res Clin Pract. (2019) 150:211–218. doi: 10.1016/j.diabres.2019.03.018, PMID: 30880089

